# Mokken scale analysis of mental health and well-being questionnaire item responses: a non-parametric IRT method in empirical research for applied health researchers

**DOI:** 10.1186/1471-2288-12-74

**Published:** 2012-06-11

**Authors:** Jan Stochl, Peter B Jones, Tim J Croudace

**Affiliations:** 1 Department of Psychiatry, University of Cambridge, Cambridge, UK; 2 CAMEO, Cambridgeshire & Peterborough Foundation NHS Trust, Cambridge, UK

## Abstract

**Background:**

Mokken scaling techniques are a useful tool for researchers who wish to construct unidimensional tests or use questionnaires that comprise multiple binary or polytomous items. The stochastic cumulative scaling model offered by this approach is ideally suited when the intention is to score an underlying latent trait by simple addition of the item response values. In our experience, the Mokken model appears to be less well-known than for example the (related) Rasch model, but is seeing increasing use in contemporary clinical research and public health. Mokken's method is a generalisation of Guttman scaling that can assist in the determination of the dimensionality of tests or scales, and enables consideration of reliability, without reliance on Cronbach's alpha. This paper provides a practical guide to the application and interpretation of this non-parametric item response theory method in empirical research with health and well-being questionnaires.

**Methods:**

Scalability of data from 1) a cross-sectional health survey (the Scottish Health Education Population Survey) and 2) a general population birth cohort study (the National Child Development Study) illustrate the method and modeling steps for dichotomous and polytomous items respectively. The questionnaire data analyzed comprise responses to the 12 item General Health Questionnaire, under the binary recoding recommended for screening applications, and the ordinal/polytomous responses to the Warwick-Edinburgh Mental Well-being Scale.

**Results and conclusions:**

After an initial analysis example in which we select items by phrasing (six positive versus six negatively worded items) we show that all items from the 12-item General Health Questionnaire (GHQ-12) – when binary scored – were scalable according to the double monotonicity model, in two short scales comprising six items each (Bech’s “well-being” and “distress” clinical scales). An illustration of ordinal item analysis confirmed that all 14 positively worded items of the Warwick-Edinburgh Mental Well-being Scale (WEMWBS) met criteria for the monotone homogeneity model but four items violated double monotonicity with respect to a single underlying dimension.

Software availability and commands used to specify unidimensionality and reliability analysis and graphical displays for diagnosing monotone homogeneity and double monotonicity are discussed, with an emphasis on current implementations in freeware.

## Introduction

Mokken scaling techniques are a scaling method that can be applied by researchers who design tests or construct multi-item questionnaires to measure health constructs [1]. Mokken scaling can also be applied as a secondary analysis approach to scrutinize the appropriateness and performance of more well established parametric item response theory (IRT) methods such as the Rasch family of models [2], which rely on stronger statistical assumptions. Further, it can be used to explore conformity with these assumptions for new data in which established items are applied to new respondent samples.

Mokken models belong to the class of statistical models called non-parametric item response theory (NIRT). They extend the simple deterministic Guttman scaling model [3] which unrealistically assumes that the data are error-free. Mokken models bring the Guttman idea within a probabilistic framework and therefore allow researchers to model data allowing for measurement error [4]. The major advantage of NIRT over more commonly used item response models, such as the Rasch model, is that they relax some of the strong (logistic ogive or sigmoid shape) assumptions about the non-linear behaviour of response probabilities that are invoked by the family of parametric IRT models [5].

**Figure 1:**
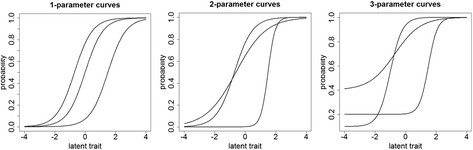
Examples of S-shaped curves for binary scored items.

More specifically, in the typical parametric approach the item characteristic curve is usually expected to follow a smooth and symmetric S-shaped function according to the family of logistic or probit cumulative distribution functions with single, 2-parameter, or more complex (3- or even 4-parameter models), see Figure 1 for examples of these curves. The smaller the number of parameters that are estimated for each item then the more restrictive the model becomes. As the number of parameters used to describe each item shape and location increases the greater the number of features in the data potentially that can be accommodated by the final scale.

An advantage of the more restrictive parametric family of IRT models is their parsimony, familiarity, and arguably, their straightforward interpretation of parameters and thus findings. But these strengths have to be tempered by the likelihood that a smaller number of items are likely to be retained in the final scale. This will occur when (too) many potentially useful items are rejected because of the shape of their item response functions, with the result that other aspects of scale performance are likely to be compromised to some extent. For example, reliability estimated from the conforming items may return values that are unsatisfactorily low.

It is important to recognize that some of the special features of the most restrictive parametric models (such as the Rasch model) are, however, not caused by the logistic or probit nature of the item characteristic curve/response function but by the requirement that the item characteristic curves (ICCs) do not intersect. A detailed comparison between the Mokken and Rasch approaches, of which we say little more here, can be found in the work of Meijer, Sijtsma, & Smid [6], to which we refer the reader for more detailed information on this particular detail.

**Table 1:** Summary table of key terms

**Key term**	**Explanation**
Mokken models	Two probabilistic models (MHM and DMM, see below) which relax strict assumptions on the shape of the ICCs imposed by traditional parametric models such as Rasch or two-parameter logistic model.
Latent trait (*θ*)	Latent construct intended to be assessed with a scale
Item	A question in a measure (linked to response category options)
Item characteristic curve (ICC)	Probability of endorsement of specific response category as a function of latent trait
Unidimensionality	Scale under consideration measures a single latent trait
Monotonicity	ICC is a monotonically increasing function, i.e the higher the value of latent trait, the higher is the ICC; actually it may be the same, but not lower.
Non-intersection	ICCs that do not intersect with each other
Monotone homogeneity model (MHM)	Mokken model assuming unidimensionality, monotonicity, and local independence of items within a scale. After these assumptions are checked, respondents can be ordered according to the simple sum score of items (at least for scales that consist of binary responses)
Double monotonicity model (DMM)	Mokken model assuming unidimensionality, monotonicity, local independence and non-intersection of items within a scale. If these assumptions are met then (additionally to MHM features) items have IIO property.
Scalability coefficient	Index of homogeneity of item pairs (*H*_*ij*_), items (*H*_*i*_) and scale (*H*) used in assessment of unidimensionality.
Invariant item ordering (IIO)	Items have the same “difficulty” ordering irrespective of value of latent trait. Consequences resulting from IIO are described in the introduction section.

Under a parametric IRT model approach the decision to keep or discard an item from the final scale is based partly on the item fit, whether informally or formally graphically assessed, or testing using, for example, a multiple degree of freedom likelihood ratio (chi-square) test (LRT). Some aspects of item misfit are often attributable to the deviation from the assumed functional form of the item regression model, which are typically logistic or normal-ogive (probit) in form. The simple notion behind the origination of NIRT is to relax this function to more general - though still monotonically increasing - form, without the degree of regularity characteristic of the sigmoid-shapes that result from the use of the logistic or probit link functions.

This broader, more inclusive approach enables items with consistent (increasing) but less regular curve shapes for the ICCs to be included. Under the condition that other assumptions (which we now introduce) are also met, this reduction of misfit can result in more items from a pool, being retained in any candidate scale. Thus inclusion of more items can reduce the likelihood of observing low scale reliability values which could characterize and compromise (in terms of usefulness) an otherwise over-shortened scale. It must be borne in mind however, that the consequence of adopting this nonparametric approach for scale building is that only ordinal information about the location of items and persons on the latent measurement continuum is available to the researcher, but not the ability estimates nor the item locations themselves [7].

Two NIRT models for dichotomous items, referred to here as the *monotone homogeneity model* (MHM) and the *double monotonicity model* (DMM), are described in the literature: both were introduced in a text by Mokken [8] over forty years ago for dichotomous (binary) items. An extension of both models for polytomous (ordinal) items was proposed by Molenaar [9] in work a decade later. For dichotomous item responses, if the data are adequately fit to the MHM then highly useful property that is ensured by these results is the ordering of respondents with respect to their latent value/”ability” on the basis of the simple sum score of their correct responses (denoted as *X*_*+*_). Another feature of the MHM is that apart from ties, the expected order of the persons on the latent measurement continuum is the same for each selection of items (provided that they are from a monotonely homogeneous item pool). In this sense, person ordering using items with monotonely non-decreasing IRFs can be thought of as "item-free“, at least in theory; a property that might be useful for applied researchers who face challenges in their research that can only be overcome by exposing different individuals to different items e.g. to avoid repeated exposure, in repeated measures studies or panel designs [7].

If the dichotomous items fit the more restrictive DMM it implies that the ordering of the items (with respect to the probability of the indicated response) is the same at all locations on the latent measurement continuum. This feature is called an invariant item ordering (IIO) [10]. IIO allows the researcher to order items according to their difficulty (facility) or commonality/prevalence, a property that helps researchers to communicate useful features of the hierachical ordering of scale items to users. This feature is particularly appreciated and has been widely exploited, for example, in intelligence or developmental tests, and also in rehabilitation outcome measurement where recovery is understood in terms of the re-acquiring, in an expected order, of tasks or functions of different levels of difficulty. This places an emphasis on the establishment of cumulative hierachical scales on a unidimensional continuum.

Scales that fit the DMM also have several other useful features: For example, if a respondent is known to have answered 6 (out) of 10 dichotomous items correctly then it is most *likely* (since the DMM is a probabilistic model) that the 6 items answered correctly were the *easiest* items in the set. In health applications this would apply to common or rare symptoms, such that rare symptoms would usually indicate common symptoms present also (if a cumulative hierarchical scale holds). In addition, if the DMM fits the item response data then the IIO property can also be expected to hold in any subgroup from the population and thus is considered to be in some sense „person-free“. One consequence of this is that if a researchers finds that the DMM does not fit the data then it might be one indication that measurement invariance may need to be considered, since this failure can result from the presence of differential item functioning [7], where the issue of IRF shape possibly differing across groups is considered. Although clearly important, wider issues of measurement invariance and DIF in NIRT are outside our scope here.

For items scored in more than just 2 categories (i.e. polytomous items) fitting the MHM does not (theoretically) imply that respondents can be ordered on the basis of their sum score *X*_*+*_[11]. However one simulation study [12] has shown that *X*_*+*_ can be used in practice as a proxy for ordering persons with respect to their latent values without risk of many serious errors. Importantly, fitting the DMM to polytomous item response data does not imply the IIO property. If this feature is desired other methods have been proposed to evaluate this aspect [13,14]. To our knowledge these methods are yet to be implemented in the commercial software MSPWin but it is useful that they have recently become freely available within package “mokken” in the freeware statistical computing environment R [15,16].

Although under NIRT the assumption regarding a sigmoid-like functional form is relaxed for the IRF, there are still further assumptions that must be met in order to permit and specify Mokken modeling. These assumptions are now introduced for the reader in the next section, which is intended as a guide for applied researchers.

### Assumption of unidimensionality

The assumption of unidimensionality means that all items from the same instrument, test or subscale i.e. the “item set” share (measure) the same latent trait (*θ*) apart from any unique characteristics of (any) item(s) and the presence of some ubiquitous measurement error. Sijtsma and Molenaar [5] formulate some interpretations of this assumption:

a) The *psychological interpretation* is that all of the items measure one thing whether an ability or another construct - for example cognitive level of respondents or their mental well-being,

b) the *mathematical interpretation* says that only one latent variable is necessary to account for the inter-item associations in the empirical data.

### Assumption of local independence

A second, equally important assumption, first defined by Anderson [17], is actually not specific to NIRT; it is required by various statistical and measurement models. Within the NIRT framework, the local independence assumption states that an individual’s response to an item *i* is not influenced by his or her responses to the other items in the same test/scale [5].

Local independence can be violated, for example, by learning through test-taking practice: this is quite possible, and is easy to imagine. Consider a situation where during testing the latent trait value of an individual test-taker (*θ*) may in fact change, increasing in level through knowledge obtained from the test itself (from the questions), or simply through repeated practice with items on a particular topic or of a particular type. Conversely a trait might also be considered to decrease, for example, because the test taker becomes tired and no longer performs the remainder of the test to the same cognitive level.

Although detecting violations of local independence is quite difficult, some statistical methods have been developed for this purpose, that enable examination of such “local item dependence” in practice, e.g. Douglas, Kim, Habing, and Gao [18], Ip [19,20] or Hoskens and De Boeck [21]. We have chosen not to discuss this issue in any detail here, since it goes beyond the intended scope of this introductory paper. Suffice to say, where there is some suspicion or more concrete hypothesis regarding local dependence the researcher is, of course, advised to perform statistical testing or to attempt to model the local dependence in some way, considering perhaps a structural equation modeling approach, where the researcher might consider it useful to correlate the measurement error residuals between the potentially dependent items.

### Assumption of monotonicity

The third assumption is that for each item the probability of a particular response level, for example in the case of a simple correct/incorrect (binary) responses, the correct answer, or endorsed response *P*_*i*_(*θ*) is a monotonically non-decreasing function of the latent trait *θ*. Investigation of this assumption is an important step in using NIRT models. Typically, the software for performing Mokken scaling analysis also provides graphical displays as well as numerical summaries to assist in this aspect of model evaluation.

### Assumption of non-intersection

The three assumptions introduced above are sufficient for many applications of NIRT. They comprise the assumptions of the monotone homogeneity model (MHM). The second and more restrictive DMM requires the additional assumption of non-intersecting of ICCs across *θ*. ICCs may touch locally and may even coincide completely in the extreme case [7].

For dichotomously scored items this assumption implies the following: if it is known that the probability of a correct answer for item *k* is lower than for item *l* for one value of *θ* and the assumption of non-intersection of ICCs holds then

(1) $$P_{k}(\theta )<P_{l}(\theta )\text{,}$$

for all values of *θ*.

Non-intersection of ICCs for dichotomously scored items ascertains an IIO of items and thus scales meeting this assumption can be considered as nonparametric counterparts to scales based on the more traditional, parametric Rasch models. For polytomously scored items with *m* response categories and *m-*1 ICCs for every item, non-intersection does not guarantee IIO (for details see [13]). Fitting DMM for polytomous items within MSPWin only orders the *m-*1 ICCs within each item, but *not* the items themselves [7] and therefore IIO is not assured. Methods for investigation of IIO for polytomous items [14,22] are available to researchers in the “mokken” package of the free software R.

### Scalability coefficients

Homogeneity coefficients play an important role in the process of Mokken scale analysis. The relevant scalability coefficients were first introduced by Loevinger [23] for the purpose of evaluation of the homogeneity of a set of items. The scalability of each item pair coefficient (*H*_*ij*_) was defined by her as the ratio of the covariance of items *i* and *j*; and the maximum covariance given the marginals of the bivariate cross-classification table of scores on items *i* and *j*, that is,

(2) $$H_{\mathrm{ij}}=\frac{Cov(X_{i},X_{j})}{Cov\max(X_{i},X_{j})}\text{.}$$

The item scalability coefficient *H*_*i*_ is defined as

(3) $$H_{i}=\frac{\sum_{j\neq i}Cov(X_{i},X_{j})}{\sum_{j\neq i}Cov_{\max}(X_{i},X_{j})}\text{,}$$

and the scalability coefficient *H* for *k* items is defined as

(4) $$H=\frac{\sum_{i=1}^{k-1}\sum_{j=i+1}^{k}Cov(X_{i},X_{j})}{\sum_{i=1}^{k-1}\sum_{j=i+1}^{k}Cov_{\max}(X_{i},X_{j})}\text{.}$$

It has been shown by Hemker, Sijtsma, and Molenaar [24] that given the MHM, all *H*_*ij*_*H*_*i*_ and *H* coefficients must take values ranging from 0 to 1.

Generally, if *H* = 1, there is no disordering or “inversion” of the item responses. If *H* = 0, this means that there is no linear relation among the test items (no correlation). Therefore, *H* can be considered as measure of the accuracy by which items within a scale are able to order the respondents [25]. Under the MHM, *H* values of 0 mean that for *k*-1 items the ICCs are constant functions of *θ*[24]. As a rule of thumb in practical interpretation of analyses, scales with *H* < 0.3 are not considered as unidimensional. Item sets with *H* coefficients higher than 0.3 and lower than 0.4 are typically considered to be indicative of only weak scales: unidimensional but not strong in any scaling sense. When *H* ranges between 0.4 and <0.5 the scales is considered of medium strength and only when *H* > 0.5, the scale is seen as strong [5]. Higher *H* values mean that the slope of the ICCs tend to be steeper, which implies that the items discriminate better among different values of *θ*[26].

For dichotomous items, if *H* is calculated on the transposed data matrix then we obtain a measure summarizing the accuracy of item ordering within a scale [27]. Such a coefficient is denoted as *H*^*T*^ and has been generalized for the polytomous case by Ligtvoet et al. [14]. These authors propose similar rules of thumb for the interpretation of *H*^*T*^: values of *H*^*T*^ below 0.3 indicate that the item ordering is too inaccurate to be practically useful; 0.3 ≤ *H*^*T*^ < 0.4 means somewhat low accuracy; 0.4 ≤ *H*^*T*^ < 0.5 represents only medium accuracy; and values over 0.5 indicate high enough (sufficient) accuracy of item ordering within a scale [14].

To assess whether an item is coherent enough to be included in the scale, the corresponding item coefficient *H*_*i*_ is used. All *H*_*i*_s in the unidimensional scale should be larger than 0.3. During the process of analysis of the scale, the cutoff value of *H*_*i*_ must be specified by the researcher. What value to choose is a decision that depends on the researcher’s need for homogeneity of items as a property of the final scale(s) – with the decision to set a higher cutoff value of *H*_*i*_, indicating the need for a higher level of homogeneity (unidimensionality).

### A method for estimating the reliability of the total score (*X*_*+*_)

Sijtsma and Molenaar [28] proposed a method to estimate the reliability of the total score *X*_+_. This method is based on some initial ideas that were proposed earlier in Mokken’s text [8] and estimates reliability as the probability of giving the same response twice. This quantity is obviously not observed but can be extrapolated and interpolated on the basis of proportion of respondents who give positive responses to item pairs [29]. Such an approach assumes that the ICCs do not intersect. Thus, before interpreting the reliability estimate it is necessary to check whether the double monotonicity model (DMM) assumptions are met, addressing any violations that are observed, in successive iterations of the procedure. The reliability estimated by this method is commonly denoted by the coefficient *Rho*. Sijtsma and Molenaar [28] showed that in number of cases the *Rho* coefficient is almost unbiased, whereas Cronbach's alpha coefficient [30] always underestimates the reliability of the total score. Readers interested in details of estimation of reliability within context of NIRT and relationship to internal consistency, Cronbach's alpha and *Rho* are referred to other sources for more information [29,31].

### Examples and interpretation of results

#### Example 1: dichotomously scored items (binary response data)

##### Step by step analysis of a short self-report screening questionnaire: Goldberg’s 12-item General Health Questionnaire (GHQ-12) with “traditional” scoring

For our first analysis, we have selected an instrument that is widely documented in the psychiatric literature, and used in range of other health care and general population settings. The GHQ-12 (see Appendix 3) was developed as a brief, self-completion (typically paper and pencil) screening questionnaire that can be used to identify groups at risk for non-psychotic psychiatric morbidity [32]. For brevity and simplicity, we have only considered the simplest scoring method for this instrument, which is recommended when the GHQ is used as a screening questionnaire. The “traditional” scoring method is dichotomization of the polytomous responses, i.e. original 1-2-3-4 scores were recorded to 0-0-1-1. Although this scoring method potentially removes some important variation in responses, there is comparatively little loss in screening efficiency by collapsing the item responses in this way, since the prevalence in the highest (4) category, is usually very low (rare), often <5%.

Other versions of this scale include the GHQ-60, GHQ-30 or the 28 item “scaled” (multidimensional) GHQ, which could also be studied using these methods [29,33,34], but here we take this opportunity to report a pedagogical analysis of the most commonly used version. We have chosen to exemplify the use of the freeware R environment [15] and the user-written package (R library) called “mokken” [16] which enables readers to replicate our analysis steps (see outlined R-code in the Appendix 2).

The potentially contentious feature of the GHQ is item wording since six of these twelve items are positively phrased and six are negatively phrased. In clinic samples, it has been established that the positively and negatively worded items constitute two separate subscales, that Bech has argued should be named “well-being” and “distress” [35]. Loevinger coefficients of homogeneity are only meaningful if the questionnaire under examination already has clinical validity [36]. We therefore present results throughout our paper (on the advice of one of the referees) for the two six item unidimensional sets corresponding to these subscales, not for the twelve GHQ items in any single analysis. Broader issues related to alternative scoring of this instrument are not considered in this pedagogical introduction to maintain the brevity and focus.

##### Sample

GHQ-12 responses analysed here (after binary recoding) are from one of the Scottish Health Education Population Survey (SHEPS) samples recruited from 1996 to 2007. We have used data from year 2006 (SHEPS Wave 19) with 355 men and 418 women (mean age 47.5 years, min = 16 years, max = 74 years).

##### Step 1 – assessment of dimensionality

Since both NIRT models assume unidimensionality of item sets we first examined whether all 12 items can be considered to measure a single underlying construct. It is suggested that Loevinger's scalability coefficients can be used for assessment of unidimensionality. The *H* and *H*_*i*_ coefficients (and also *H*_*ij*_*)* are obtained using function *coefH,* after installing the mokken package in R and loading its command library. For these GHQ data, the values for the *H*_*i*_ coefficients are shown in Table 2, for the two scales formulated by Bech et al. [33] (“well-being” and “distress”).

**Table 2:** ** *H* **_** *i* **_**coefficients for binary recoded GHQ items (1-2-3-4 to 0-0-1-1, i.e. traditional scoring method)**

	
**Wellbeing:**	
Label	*H*_*i*_
	*(item scalability)*
Concentrate	0.57
Useful	0.51
Make decisions	0.62
Enjoy	0.52
Face problems	0.58
Reasonably happy	0.54
Distress:	
Label	*H*_*i*_
	*(item scalability)*
Lost sleep	0.58
Under Strain	0.67
Overcome	0.61
Unhappy	0.64
Losing confidence	0.64
Worthless	0.68

Here we consider these first results from our Mokken scaling analysis. First we observe that none of the *H*_*i*_ coefficients fall below the suggested threshold level of 0.3 for either subscale, which was recommended earlier, from available guidance in the literature, as a level worthy of retaining items. This pattern of results, and *H*_*i*_ values across items suggest that, in this population sample, all of the GHQ-12 questionnaire items *within their respective subscale* are sufficiently homogeneous (correlated) enough to comprise a separate scales and thus measure underlying constructs (well-being scores versus distress scores, to use terminology from [33]). Also, the summary *H* coefficients, here equal to 0.55 (well-being) and 0.63 (distress), suggests that both subscales are homogeneous enough to be considered as unidimensional measures.

The Mokken package also features algorithms (implemented in the function called *aisp*) for automated search of unidimensional scales (item sets) from the item pool. This is effectively a method for executing the same “search” option that is available in the commercial package MSPWin that we will introduce to the reader in the polytomous items analysis reported below as our second example.

##### Step2 – assessment of monotonicity

Next we consider the assessment of monotonicity, i.e. that item characteristics curves are monotonically increasing functions of latent trait. Monotonicity is an important feature since it allows the researcher to order respondents on a latent continuum with respect to the sum score of the items belonging to the same scale. In practice monotonicity of item *i* is assessed by replacing the unobserved latent trait value with a *restscore* (which is simply the sum score of all of the items except for item *i*). If monotonicity holds, then apart from sampling fluctuation it can be expected that in the group of persons with restscore *s* a higher proportion will endorse the item step than in the group of persons below with restscore *r,* for any pair of numbers *r* < *s*. If in any pair this predicted order is reversed, this is referred to as a scaling violation [7]. These violations are counted and displayed for inspection by the user in both MSPWin and R program output.

The monotonicity requirement can be assessed by calling function *check.monotonicity* in R. There are two different settings regarding monotonicity that can be set by the researcher. The first one is the minimum size of the restscore group (denoted as the *minsize*). If the adjacent restscore groups contain less than a chosen value for the minimum size, then they are joined together. This feature is intended to avoid some unstable proportion estimates which would otherwise potentially obscure the assessment of monotonicity. However, increasing the *minsize* (especially for small datasets with low sample sizes) to higher values may in turn lead to the presence of too few restscore groups with the consequence that the monotonicity requirement then cannot be meaningfully assessed. Small values of *minsize* lead to many restscore groups but when the sample size is small the number of individuals within each restscore group may become small and thus violations of monotonicity may occur simply due to sampling error. Therefore, a sensible strategy for small sample datasets is to set up the minimum size of restscore groups in a way that leads to 4 restscore groups. The default setting for *minsize* in package “mokken” is as follows [16]: If we denote N as sample size then *minsize* = N/10 if N > 500; *minsize* = N/5 if 250 < N < 500; and *minsize* = N/3 (but at least 50) if N < 250.

The second setting that can be set by researchers is denoted as the *minvi*. It is a minimum value for the decrease of the IRF (or the ISRF if the data being analyses are polytomous) that is necessary to be counted as violation of monotonicity. Increasing this number leads to necessarily fewer violations. Increasing the *minvi* value might be a wise strategy for small samples where decrease may be due to sampling error.

The results of monotonicity assessment (from the R program) are displayed in Table 3.

**Table 3:** Abridged output of assessment of monotonicity


**Wellbeing:**			
Label	#ac	#vi	#zsig
	(#active comparisons)	(#violations)	(#significant violations)
Concentrate	6	0	0
Useful	6	0	0
Make decisions	6	0	0
Enjoy	6	0	0
Face problems	6	0	0
Reasonably happy	6	0	0
Distress:			
Label	#ac	#vi	#zsig
	(#active comparisons)	(#violations)	(#significant violations)
Lost sleep	6	0	0
Under Strain	6	0	0
Overcome	6	0	0
Unhappy	6	0	0
Losing confidence	6	0	0
Worthless	6	0	0

**Figure 2:**
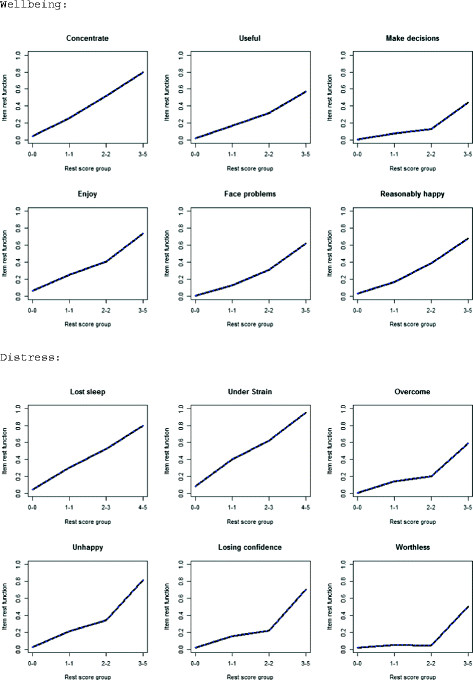
Monotonicity plots of GHQ-12 items under traditional (binary 0-0-1-1) scoring.

Here, #ac indicates the number of active comparisons that are checked in the data for monotonicity violation. Its value depends on the number of restscore groups. Because we have 4 restscore groups (see Figure 2) the number of active pair comparisons is (4 × 3)/2 = 6 (see [7] for more details). These results indicate that there are no significant (#zsig) and not even any nonsignificant (#vi) violations of monotonicity for any of the GHQ-12 items within subscales. In other words, all items appear to discriminate well between respondents with high levels on the construct (expressed as high restscore) and ones with lower levels (low restscore).

Monotonicity plots are available in R by using the function *plot* and also in MSPWin software. We present them to offer a more detailed understanding of what monotonicity means: the violation of monotonicity of the item means that the corresponding curve would not be monotonically increasing but would show some local “decrease” at least of the value specified in *minvi*. Figure 2 shows what has been already summarized in the entries for Table 3, i.e. that there are no violations of monotonicity, since all curves are monotonically increasing.

##### Step 3 – assessment of non-intersection and IIO

For dichotomously scored items non-intersection of item characteristics curves (ICCs) ascertains IIO and this can be investigated either in R or in MSPWin. The most recently developed procedures [14,22] are freely available in the R library “mokken”. Additional functionality includes a method for selection of items that together compose a scale having IIO as well as reporting an *H*^*T*^ coefficient.

Checking of IIO within the R “mokken” library is done using the function *check.iio*. Three different method options are available to users – (1) manifest invariant item ordering (MIIO), (2) manifest scale - cumulative probability mode (MSCPM) and (3) increasingness in transposition (IT). The MIIO has been chosen in this example analysis simply because no detailed description of the MSCPM and IT options was available to the author at the time of submission of this manuscript.

The backward selection method can be used to remove items violating IIO. If there is an equal number of violations for two or more items then the item with the lowest scalability is removed [14]. As an alternative to this exploratory approach, the worst item found to be violating the IIO property can be discarded and IIO of the rest of the items checked again, in iterative steps. Likewise for monotonicity, it is recommended to only remove one item at a time since IIO violations of other items may be influenced by the inclusion or exclusion of any particular item.

Table 4 shows R results of IIO investigation for both subscales of the GHQ-12. No violations of the invariant item ordering requirement are present.

**Table 4:** Abridged output of assessment of IIO


**Wellbeing:**				
Label	Mean	#ac	#vi	#tsig
		(#active comparisons)	(#violations)	(#significant violations)
Wellbeing:				
Concentrate	0.17	9	0	0
Useful	0.11	10	0	0
Make decisions	0.07	10	0	0
Enjoy	0.17	9	0	0
Face problems	0.10	10	0	0
Reasonably happy	0.13	10	0	0
Distress:				
Label	Mean	#ac	#vi	#tsig
		(#active comparisons)	(#violations)	(#significant violations)
Lost sleep	0.21	10	0	0
Under strain	0.26	10	0	0
Overcome	0.13	10	0	0
Unhappy	0.18	10	0	0
Losing confidence	0.15	10	0	0
Worthless	0.10	10	0	0

We can therefore consider the results of this first exemplar Mokken scaling analysis as supportive of a GHQ-12 scale which, within each subscale, satisfies IIO and therefore both subscales have the properties described in the introduction. None of the GHQ-12 items need to be deleted under the assumptions of this scaling model.

In the following more complex analysis example, we consider data from a different instrument, the WEMWBS which has only positively worded items but 14 five category Likert-style response items, i.e. polytomous responses.

#### Example 2: polytomous response data

##### Step by step analysis of the Warwick-Edinburgh Mental Well-being Scale (WEMWBS)

We now present a second empirical analysis example using a different instrument for which the intended underlying measure is a population-wide rating scale for mental well-being. WEMWBS (see Appendix 4) comprises 14 items scored in one of five ordinal Likert-style response categories, which uses the same verbal anchors for each item. This type of numerical rating scale is traditionally scored from 1 to 5 or zero to 4. We have chosen a relatively new measure, developed to measure positive well-being in populations, since there is currently a lot of interest in this area in a wide range of disciplines from economics through to epidemiology, and the issue of measurement (validity and reliability) is of utmost importance for the credibility of empirical claims in the field, and also for policy evaluation by governments. Elsewhere, items from this scale have been subjected to parametric IRT analysis (using the Rasch model) [37,38].

Our aim once again is to describe the analysis in logical steps to illustrate how applied researchers might apply the technique of Mokken scaling to ordinal responses, but this time in the MSPWin software environment, a dedicated NIRT program (although the same can be obtained within R).

##### Sample

In contrast to our first example, where the data were from a cross-sectional survey, data for this example the data come from a major longitudinal British cohort study (initiated in 1958), the National Child Development Study (NCDS; UK Data Archive Study SN6137). This sample, being from a birth cohort, is homogeneous for age (in years), at the time of assessment. At age 53, 8643 NCDS survey members (4168 men and 4475 women) completed the WEMWBS items as part of a set of self-completion questionnaires.

##### Step 1 – assessment of dimensionality

Since both the monotone homogeneity model (MHM) and the double monotonicity model (DMM) assume unidimensionality of item sets we first examined whether all 14 of the WEMWBS items do indeed measure a single dimension of mental well-being, as intended. In the case of the WEMWBS, the underlying construct purported to be measured is considered to be *mental well-being*, an aspect of positive mental health that might start from signs of mental-ill health, but spans also more positive states.

Similarly to our first example, Loevinger's scalability coefficients are suitable as a measure of homogeneity of items (*H*_*i*_ coefficients) and scale (*H* coefficient). There are two options for assessing unidimensionality available in commercial software MSPWin. These are presented in the next two sections.

##### Option 1

The first evaluation approach uses the “test option” in MSPWin. Under this procedure for all items under analysis, item scalability coefficients (*H*_*i*_) are computed. If all the *H*_*i*_ values are over the recommended lower bound cutoff for a unidimensional scale (the threshold being 0.3) then the researcher may safely treat the scale as unidimensional and proceed to step 2. If not it is advised to remove the item with the lowest *H*_*i*_, and then re-run the analysis to check whether the *H*_*i*_ values of the other items improve. This strategy can be repeated until one is satisfied that only items confirming to an unidimensional scale remain in the analysis.

If more than a minority of items are discarded then it is prudent to consider rejected items as a set, and these items should be checked again for unidimensionality with the same strategy just described since these items may form a scale too. It is sensible to consider this set as a new candidate scale, if face and construct validity evidence can be marshalled in support of this potential second latent continuum, and it can be interpreted in a nomological network of construct validity data, a requirement that should also be met for the primary item set dimension, but which we have neglected to emphasis so far.

For the WEMWBS items applying the MSPWin “test option” leads to the results shown in Table 5.

**Table 5:** Abridged output of test option of all items of WEMWBS

**Label**	**Mean**	**ItemH**(***H***_***i***_**)**	**Monot.**
Optimistic	3.27	0.45	0
Useful	3.56	0.47	0
Relaxed	3.30	0.50	0
Interested in others	3.54	0.33*	0
Spare energy	2.81	0.41	0
Deal with problems	3.59	0.50	0
Think clearly	3.71	0.54	0
Feel good	3.39	0.58	0
Feel close	3.58	0.47	0
Confident	3.46	0.58	0
Can decide	3.96	0.47	0
Feel loved	3.91	0.42	0
Interested	3.60	0.47	0
Cheerful	3.58	0.57	0

Scale H = 0.48.

The worst item is marked by an asterisk where relevant.

In order to reduce the number of tables we have included in Table 5 additional column (headed - Monot.) that we will not review now, but will consider later once the item selection steps (described here) have progressed further.

All *H*_*i*_ values of the items (in MSPWin these are denoted as ItemH, see column headings) were found to be above the suggested lower bound cutoff value of 0.3. This is reassuring since the scale was developed to measure only one construct, that of mental wellbeing. However, it can be observed that item *Interested in others* has a substantially lower *H*_*i*_ value than the other WEMWBS items and it is only slightly above the level of 0.3. This may be an indication that this item is more distinctive, and less strongly consistent with the content of the remainder: when *H*_*i*_ values in this range are observed, one suggestion for a practical step in the analysis is that we could remove this item, and recompute *H*_*i*_ values for the remaining set of items.

It is of interest then to see what happens when we drop item *Interested in others* from the current analysis. These results are displayed in Table 6. We notice that all *H*_*i*_s are now higher than in the previous analysis (compared to column entries in Table 5 with all 14 items). This is because the inclusion of item *Interested in others* negatively affected the magnitude of the observed values for the other item *H*_*i*_ values. Once this item was discarded the overall *H* coefficient from consideration of all items (reported as the Scale H value at the bottom of the table) increased by 0.03, from a value of 0.48 to 0.51.

**Table 6:** Abridged output of test option of WEMWBS without item 4

**Label**	**Mean**	**ItemH**(***H***_***i***_**)**	**Monot.**
Optimistic	3.27	0.46	0
Useful	3.56	0.48	0
Relaxed	3.30	0.52	0
Spare energy	2.81	0.42*	0
Deal with problems	3.59	0.52	0
Think clearly	3.71	0.56	0
Feel good	3.39	0.61	0
Feel close	3.58	0.47	0
Confident	3.46	0.60	0
Can decide	3.96	0.49	0
Feel loved	3.91	0.43	0
Interested	3.60	0.48	0
Cheerful	3.58	0.60	0

Scale H = 0.51.

The worst item is marked by an asterisk where relevant.

Now the WEMWBS scale comprising of the remaining 13 items looks to be sufficiently unidimensional in this age homogenous sample: other NIRT features can now be investigated.

##### Option 2

The second option available to researchers for considering the notion of (sufficient) unidimensionality is much less time consuming in practice but provides similar results. The researcher may use MSPWin to set a cutoff for *H*_*i*_ values and allow the program to automatically “search” for a set of items by considering alternative unidimensional scales (a situation the authors find rather hard to envisage, but nevertheless, it may exist!). Within this approach researchers are advised to run a set of analyses with different cutoffs selected for the critical *H*_*i*_ value that is applied each time.

One recommended strategy is to start with the established cutoff value of 0.3 and to increase its level subsequently in steps of say 0.05 or 0.10 to 0.50 or 0.55, observing how the items cluster into sets or scales at these alternative thresholds. By executing multiple analyses in this sequence, such a strategy can provide important insights into the relationships among items.

To provide flexibility the cutoff for *H*_*i*_ can be changed prior to analysis in the MSPWin program options. Previous experience (of the authors) shows that for cutoff values of 0.40 or 0.45 one often gets results similar to those from a parametric IRT analysis [39]. Clearly it would be foolish to advise researchers to blindly use the recommended cutoff of value 0.3 as a critical threshold. Rather, as in all cases of statistical modeling, judgment is required. It may indeed be useful to increase this cutoff if the scales obtained under higher cutoff values give more meaning (higher face validity, or provide better construct definition). To make such a judgment it is necessary to consider the theoretical rationale that lay behind the development of the instrument and which presumably underpinned the original formulation of the item contents.

Although it is always possible to expect a stronger scale (*H* values) from a higher cutoff, increasing the cutoff value above a certain level will likely lead to the possibility of many item sets (scale fragments) with only a few items. This situation is clearly most undesirable, in the sense of constructing a single new scale, but the procedure does provide insights to the researcher, down to the level of item triplets and pairs (which cannot really be scales, but might reflect what in parametric modeling would be captured by the term “minor factors”). Researchers must therefore find a balance between the length and internal structure as revealed by the exploratory analysis with different cut-points.

In this example dataset, the two strategies, using the search option versus the cutoff of 0.3, lead – as one would expect – to exactly identical results (already shown in Table 5) since no item *H*_*i*_ was found below the value of 0.3. Similarly, increasing the cutoff value for *H*_*i*_ to levels of 0.35 and 0.40 also leads to identical results to those reported in Table 6. As the reader will now be able to see for themselves, increasing the *H*_*i*_ cutoff to 0.45 results in WEMWBS item *spare energy* being the item that is identified as a potential target for removal from the 13 item set.

**Table 7:** **Abridged output of search option for *H* **_** *i* **_**cutoff of 0.45**

**Label**	**Mean**	**ItemH**(***H***_***i***_)	**Monot.**
Optimistic	3.27	0.46	0
Useful	3.56	0.49	0
Relaxed	3.30	0.51	0
Deal with problems	3.59	0.53	0
Think clearly	3.71	0.57	0
Feel good	3.39	0.61	0
Feel close	3.58	0.48	0
Confident	3.46	0.61	0
Can decide	3.96	0.51	0
Feel loved	3.91	0.45*	0
Interested	3.60	0.48	0
Cheerful	3.58	0.60	0

Scale H = 0.53.

The worst item is marked by an asterisk where relevant.

As a final step of the assessment of dimensionality we present an analysis for an even higher threshold for the cutoff value, equal to 0.5. Table 8 shows that, at this level, two item sets (scales) are found. One consists of 8 items, and therefore might be a candidate scale, whereas the second one comprises of only 2 items. In this step, four WEMWBS items are discarded (automatically by the program) since they do not belong to either of these two item sets nor create their own item set of this „strength“.

**Table 8:** **Abridged output of search option for *H* **_** *i* **_**cutoff of 0.50**


Scale 1:			
Label	Mean	ItemH(*H*_*i*_)	Monot.
Useful	3.56	0.51*	0
Relaxed	3.30	0.58	0
Deal with problems	3.59	0.59	0
Think clearly	3.71	0.65	0
Feel good	3.39	0.67	0
Confident	3.46	0.67	0
Can decide	3.96	0.57	0
Cheerful	3.58	0.63	0
Scale H = 0.61			
Scale 2:			
Label	Mean	ItemH(*H*_*i*_)	Monot.
Feel close	3.58	0.64*	0
Feel loved	3.91	0.64	0

Scale H = 0.64.

The worst item is marked by an asterisk where relevant.

The second scale consists of the discarded items from the first item set that are homogeneous enough (since we set the cutoff value at 0.50, all item *H*_*i*_s must be over this value). In general, a scale consisting of such a small number of items is not of much practical use, as a set of items, even though their common item content may be of interest (for developing further/new items, or for identifying other response features common to these items). Quite often this can isolate bloated specific items that are the same content with highly similar wording, which should be avoided, in favor of items from similar domains that are not simply linguistic reformulations of other item wording. Once again, from the authors’ experience, it is certainly common for some mental health items involving somatic symptoms of worry, or sleep problems to cluster in pairs or triplets, and this might also hold in measures of mental wellbeing.

We see here that the second scale provides only information that items *feel close* and *feel loved* correlate. We cannot conclude that the item pair forms a scale, despite the program output listing it as Scale 2; it is simply the left over set or pair remaining once the larger scale has been assembled.

Overall the results from these analyses suggest that a subset of WEMWBS items can define a unidimensional scale with no obvious sub-domains and with adequate “discrimination” of items. The only item that discriminates less strongly appears to be item *Interested in others*. By excluding this item we will get slightly “stronger” scale, as reflected in the Scale *H* values in Tables 5, 6, and 7. However we may also lose information about part of the measured domain that might be useful or important. It is important to consider the value of every discarded item in case it indicates a domain of interest and sufficient importance for which future work could hypothesis more (newly written) items.

The final decision about exclusion of items should now be based on substantive reasons not merely through casual acceptance of arbitrary quantitative values as candidate threshold levels. At this point researchers should try to ask themselves questions such as: what is the content validity of each of this discarded item? Is the item correctly formulated in its wording or sense-making? Is it understandable for respondents? Does the item provide important information that cannot be lost because losing this information will harm our insight into an aspect of a measured domain, or compromise a key aspect of content validity (for which a newly written item covering the same area, but worded differently, might be required)? Does the distribution of this item’s responses have enough variance (if the vast majority of respondents use the same response category then the variance of the responses is low and so is its covariance, correlation and homogeneity assessment against other items)?

In the presented example the inclusion or exclusion of item *Interested in others* with regards to unidimensionality is inconclusive based on this one study. On the one hand it meets criteria of *H*_*i*_ for inclusion. On the other hand this item seems to be inferior to others. Perhaps a wise strategy would be to keep that item for now and see whether it is excluded by other criteria, especially monotonicity and non-intersection, that we now go on to consider in more detail for these data.

##### Step2 – assessment of monotonicity

The idea behind the monotonicity assessment described next is the same for dichotomous and polytomous responses. The only difference is that the monotonicity applies to the item *step* response function (ISRF) and not to the item response function (IRF) as is the case for dichotomous response data.

For large datasets the software MSPWin sets *minsize* automatically. For *minvi* the default setting is 0.03, which is recommended unless the sample size is small. Also, the significance level *alpha* can be set to any value desired by the researcher, a feature which is not possible in the current software implementation available to users of R.

The summary results for this examination of violations of monotonicity for each item can be seen in the output already presented in Tables 5, 6, 7 and 8: we deliberately avoided commenting on the monotonicity column earlier in the manuscript, but now consider the information reported therein. Note that MSPWin displays blanks or “empty” values if no violations are observed. For better clarity we have replaced empty values with zeros in the subsequent tables. Detailed inspection of monotonicity is also provided among the output options available from the MSPWin program. Results for the WEMWBS with 14 items in the set are displayed in Table 9.

**Table 9:** Abridged (detail) report of monotonicity

**Label**	**ItemH**	**#ac**	**#vi**	**#zsig**	**crit**
	(***H***_***i***_**)**	**(#active comparisons)**	**(#violations)**	**(#significant violations)**	
Optimistic	0.45	112	0	0	0
Useful	0.47	112	0	0	0
Relaxed	0.50	112	0	0	0
Interested in others	0.33	112	0	0	0
Spare energy	0.41	112	0	0	0
Deal with problems	0.50	112	0	0	0
Think clearly	0.54	74	0	0	0
Feel good	0.58	136	0	0	0
Feel close	0.47	112	0	0	0
Confident	0.58	113	0	0	0
Can decide	0.47	103	0	0	0
Feel loved	0.42	112	0	0	0
Interested	0.47	112	0	0	0
Cheerful	0.57	125	0	0	0

Findings in Table 9 suggest that there are no violations of monotonicity for any of the items in the dataset as indicated by #vi values. If they are violations of monotonicity, then their seriousness can be assessed via consideration of the *crit* statistic. Molenaar & Sijtsma [7] offer guidance, suggesting that items for which the *crit* statistic falls below 40 can be considered as *not* seriously violating monotonicity requirements and thus can be safely included in any Mokken scale. However, if the violation is serious (>40) then the recommended strategy is to remove the most serious (highest violaton) item and rerun the analysis procedure, checking that a new serious violation does not occur with another item. This strategy is recommended because the number of violations can be influenced by other items in the scale, since as each item is dropped the (remaining) restscore is necessarily affected. Removing one item can decrease the number of monotonicity violations over all remaining items.

There are more statistics available in the MSPWin software report on monotonicity. Interested readers are encouraged to consult further detail provided in the comprehensive manual for the MSPWin program, in order to gain further sensitivity to these values and interpretation.

##### Step 3 – assessment of non-intersection and IIO

We now arrive at what is altogether a more challenging issue, the assessment of non-intersection and invariant item ordering (IIO). For scales consisting of polytomous items such as WEMWBS the MSPWin software cannot currently be used for the assessment of non-intersection, since only the characteristic curves *within* each item, but not the items themselves, are tested for nonintersection. As we mentioned in the section on assessment of IIO for the GHQ analysis, this important aspect of Mokken analysis is currently only possible in the functionality of the R software implementation of Mokken scaling procedures.

A summary of IIO violations for all WEMWBS items (produced in R from “mokken”) is displayed on Table 10. The values in the last column represent number of significant violations. Item *Interested in others* (not surprisingly the “worst” item with regards to our earlier investigation of homogeneity) has the most serious violations.

**Table 10:** Abridged summary per item report of IIO

**Label**	**Mean**	**#ac**	**#vi**	**#tsig**
		**(#active comparisons)**	**(#violations)**	**(#significant violations)**
Optimistic	2.27	104	2	2
Useful	2.56	106	4	4
Relaxed	2.30	105	2	2
Interested in others	2.54	104	10	10
Spare energy	1.81	104	0	0
Deal with problems	2.59	104	2	2
Think clearly	2.71	105	1	1
Feel good	2.39	105	3	3
Feel close	2.58	104	1	1
Confident	2.46	104	4	4
Can decide	2.96	105	0	0
Feel loved	2.91	104	0	0
Interested	2.60	106	2	2
Cheerful	2.58	104	5	5

Consistent with our advice above, this item is removed, and the remaining items are re-analyzed. There is no cut-off for deciding upon an acceptable number of significant violations. Therefore this process can be repeated until there are no significant violations that are present. This process can be done automatically with commands within the “mokken” R package. The results of this analysis are displayed in Table 11.

**Table 11:** Backward stepwise removal of items violating IIO

**Label**	**Step 1**	**Step 2**	**Step 3**	**Step 4**	**Step 5**
Optimistic	2	2	1	0	0
Useful	3	2	1	1	0
Relaxed	2	2	1	0	0
Interested in others	8	NA	NA	NA	NA
Spare energy	0	0	0	0	0
Deal with problems	2	1	1	1	0
Think clearly	1	0	0	0	0
Feel good	3	2	2	NA	NA
Feel close	1	0	0	0	0
Confident	4	3	NA	NA	NA
Can decide	0	0	0	0	0
Feel loved	0	0	0	0	0
Interested	1	0	0	0	0
Cheerful	3	2	2	2	NA

*H*^*T*^ = 0.31.

The values in Table 11 represent the number of violations and *NA*s indicate that the item has been removed. Thus in the second step item *Interested in others* has been removed, in the third step item *Confident* and so on. After removal of four items (item 4, 10, 8 14) there are no more significant violations of IIO. The *H*^*T*^ coefficient for the final subset of items equals to 0.31, a value slightly above the minimum useful threshold for IIO proposed by Ligtvoet et al. [14].

Our polytomous item Mokken scaling analysis therefore confirms that these remaining 10 items compose a scale having IIO properties. Such a scale can be considered as satisfying the strong Mokken double monotonicity model, which was the goal of our non-parametric item response theory analysis here. These analyses exemplify what applied researchers should consider when applying mokken scaling in practice.

We suggested that one of the reasons to use NIRT is that more items might be able to be retained in the final scale, comparing to traditional parametric approaches, which make harder demands on items. For comparison therefore, it is useful to be able to consider results presented here with those from a parametric IRT/Rasch analysis already performed on ordinal response data to the WEMWBS scale items [37]. The results reported in that study, by the instrument developers, resulted in only 7 items being retained, and recommended as a WEMWBS shortform. A more detailed comparison of the results from both studies reveals that all items rejected from final 10 item scale in the presented sample were also rejected from the shortform WEMWBS-7. Those 3 additional items rejected under the parametric IRT approach but retained in our non-parametric IRT methodology can be viewed as an advantage in favour of considering the weaker (more flexible) ICC properties, and a benefit of non-parametric IRT modeling.

Unfortunately, the authors of the parametric analysis of WEMWBS used only the Rasch partial credit model which limits direct comparison of the main psychometric features with our non-parametric approach. A subtle point important to highlight at this juncture is the ability to contrast these two models: two features of the models, stochastic ordering based on *X*_*+*_ and IIO, are actually not comparable between these alternative models [40]. To be clear, stochastic ordering based on the sum score *X*_*+*_ is assured for the parametric partial credit model (and therefore for the current SWEMWBS) but not for the DMM, and therefore not for our 10 item subset of WEMWBS). On the other hand IIO is assured for our scale but not for the shortform WEMWBS-7.

The WEMWBS-7 is now being used in the field, but it seems possible that it has been over-shortened. Our conclusion is that under a non-parametric DMM model it is possible to retain three more items. Since this is almost half as many items again, there will be a consequent increase in reliability.

In closing, we recommend to applied researchers that they consider not only the opportunity to apply parametric IRT and non-parametric IRT models, but also begin to further understand their ramifications. Errors in psychometric interpretation have been identified in recent applied papers. These have been corrected by psychometricians [41], and a future dialogue between psychometricians and applied researchers on the relative importance of these versatile models is encouraged as well as wide access to practical tools for training and model evaluation.

## Appendix 1

### Available software

a) Commercial software MSPWin, currently in version 5

This software provides assessment of unidimensionality via scalability coefficients, construction of scales based on scalability coefficients, statistics for assessment of monotonicity, 3 methods for assessment of non-intersection, investigation of IIO for dichotomous items and estimation of reliability of the scales. Does not provide investigation of IIO for polytomous items.

b) Freely available R software and library “mokken”

Provides all basic statistics as MSPWin plus investigation of IIO for polytomous items including backward selection of items satisfying IIO.

c) Commercial software Stata (mokken and msp commands; [43,44])

Provides construction of scales based on scalability coefficients, statistics for assessment of monotonicity and non-intersection, investigation of IIO for dichotomous items. Does not provide investigation of IIO for polytomous items.

## Appendix 2

Outline of the R code.

#Example 1 (GHQ-12)

# In the following code “ghqdich” should be replaced by the name of the dataset imported to R

library(mokken) #opens Mokken library

scales < − aisp(ghqdich, search = "normal", lowerbound = .3, alpha = .05, popsize = 20, verbose = TRUE) #automated searching for unidimensional scales

coefH(ghqdich) #prints *H*, *H*_*i*_ and *H*_*ij*_*c*oefficients

monotonicity < − check.monotonicity(ghqdich, minvi = 0.03, minsize = 77) # assessment of monotonicity

summary(monotonicity) #summary for assessment of monotonicity

plot(monotonicity) #plots from Figure 2

iio < − check.iio(ghqdich, method = "MIIO", minvi = 0.18, minsize = 77, alpha = .05, item.selection = TRUE, verbose = TRUE) #assessment of invariant item ordering

summary(iio) # summary for assessment of invariant item ordering

check.reliability(ghqdich) #prints reliability coefficients

#Example 2 (WEMWBS)

# In the following code “wemwbs” should be replaced by the name of the dataset imported to R

iio < − check.iio(wemwbs, method = "MIIO", minvi = 0.18, minsize = 863, alpha = .05, item.selection = TRUE, verbose = TRUE) # assessment of invariant item ordering

summary(iio) # summary for assessment of invariant item ordering

## Appendix 3

### Wording of the 12-item General Health Questionnaire (GHQ-12) items

1. Able to concentrate [Concentrate]

2. Lost sleep over worry [Lost sleep]

3. Play a useful part [Useful]

4. Capable of making decisions [Make decisions]

5. Constantly under strain [Under strain]

6. Couldn’t overcome difficulties [Overcome]

7. Enjoy normal activities [Enjoy]

8. Face up to problems [Face problems]

9. Unhappy and depressed [Unhappy]

10. Losing confidence in yourself [Losing confidence]

11. Thinking of yourself as worthless [Worthless]

12. Feeling reasonably happy [Reasonably happy]

## Appendix 4

### Wording of the Edinburgh Mental Well-being Scale (WEMWBS) items

1. I’ve been feeling optimistic about the future [Optimistic]

2. I’ve been feeling useful [Useful]

3. I’ve been feeling relaxed [Relaxed]

4. I’ve been feeling interested in other people [Interested in others]

5. I’ve had energy to spare [Spare energy]

6. I’ve been dealing with problems well [Deal with problems]

7. I’ve been thinking clearly [Think clearly]

8. I’ve been feeling good about myself [Feel good]

9. I’ve been feeling close to other people [Feel close]

10. I’ve been feeling confident [Confident]

11. I’ve been able to make up my own mind about things [Can decide]

12. I’ve been feeling loved [Feel loved]

13. I’ve been interested in new things [Interested]

14. I’ve been feeling cheerful [Cheerful]

Warwick-Edinburgh Mental Well-being Scale (WEMWBS) © NHS Health Scotland, University of Warwick and University of Edinburgh, 2006, all rights reserved

## Competing interests

The author(s) declare that they have no competing interests.

## Authors’ contribution

JS performed the analyses and drafted the first version of the manuscript. PBJ reviewed the manuscript. TJC nominated and obtained the datasets used for the practical examples, helped to draft, review and improve the manuscript and provide substantive interpretation of the analyses. All authors read and approved the final manuscript.
